# Investigation of Cervical Tumor Biopsies for Chromosomal Loss of Heterozygosity (LOH) and Microsatellite Instability (MSI) at the *HLA* II Locus in HIV-1/HPV Co-infected Women

**DOI:** 10.3389/fonc.2019.00951

**Published:** 2019-10-15

**Authors:** Ramadhani Chambuso, Evelyn Kaambo, Lynette Denny, Clive M. Gray, Anna-Lise Williamson, Monika Migdalska-Sęk, Gloudi Agenbag, George Rebello, Raj Ramesar

**Affiliations:** ^1^MRC Unit for Genomic and Precision Medicine, Division of Human Genetics, Department of Pathology, Faculty of Health Sciences, University of Cape Town, Cape Town, South Africa; ^2^Department of Gynaecology, Morogoro Regional Referral Hospital, Morogoro, Tanzania; ^3^Division of Medical Virology, Department of Pathology, Faculty of Health Sciences, University of Cape Town, Cape Town, South Africa; ^4^Department of Biochemistry and Medical Microbiology, University of Namibia School of Medicine, Windhoek, Namibia; ^5^South African Medical Research Council, Clinical Gynaecological Cancer Research Centre, University of Cape Town, Cape Town, South Africa; ^6^Department of Obstetrics and Gynaecology, Groote Schuur Hospital, University of Cape Town, Cape Town, South Africa; ^7^Institute of Infectious Disease and Molecular Medicine, University of Cape Town, Cape Town, South Africa; ^8^Division of Immunology, Department of Pathology and National Health Laboratory Service, University of Cape Town and Groote Schuur Hospital, Cape Town, South Africa; ^9^Department of Biomedicine and Genetics, Medical University of Lodz, Lodz, Poland

**Keywords:** cervical cancer, loss of heterozygosity, microsatellite instability, *HLA* II locus, HIV-1/HPV co-infection, host molecular genetics, genetic alterations

## Abstract

**Background:** A subgroup of women who are co-infected with human immunodeficiency virus type 1 (HIV-1) and human papillomavirus (HPV) progress rapidly to cervical disease regardless of high CD4 counts. Chromosomal loss of heterozygosity (LOH) and microsatellite instability (MSI) are early frequent genetic alterations occurring in solid tumors. Loss of an allele or part of a chromosome can have multiple functional effects on immune response genes, oncogenes, DNA damage-repair genes, and tumor-suppressor genes. To characterize the genetic alterations that may influence rapid tumor progression in some HIV-1-positive women, the extent of LOH and MSI at the *HLA* II locus on chromosome 6p in cervical tumor biopsy DNA samples with regard to HIV-1/HPV co-infection in South African women was investigated.

**Methods:** A total of 164 women with cervical disease were recruited for this study, of which 74 were HIV-1-positive and 90 were HIV-1-seronegative. DNA from cervical tumors and matched buccal swabs were used for analyses. Six fluorescently-labeled oligonucleotide primer pairs in a multiplex PCR amplification were used to study LOH and MSI. Pearson chi-squared test for homogeneity of proportions using an exact *p* value, a two-proportion Z-score test, ROC curves and a logistic regression model were used for statistical analyses. All *p*-values were corrected for false discovery rate (FDR) using the Benjamini-Hochberg test and the adjusted *p*-values (*q*-values) were reported. All tests were significant when both p and *q* < 0.05.

**Results:** Tumor DNA from HIV-1/HPV co-infected women demonstrated a higher frequency of LOH/MSI at the *HLA* II locus on chromosome 6p21.21 than tumor DNA from HIV-1-seronegative women (D6S2447, 74.2 vs. 42.6%; *p* = 0.001, *q* = 0.003), D6S2881 at 6p21.31 (78.3 vs. 42.9%; *p* = 0.002, *q* = 0.004), D6S2666 at 6p21.32 (79 vs. 57.1%; *p* = 0.035, *q* = 0.052), and D6S2746, at 6p21.33 (64.3 vs. 29.4%; *p* < 0.001, *q* < 0.001), respectively.

**Conclusions:** HPV infection alone can induce LOH/MSI at the *HLA* II locus in cervical tumor DNA, whereas HIV-1 co-infection exacerbates it, suggesting that this may accelerate cervical disease progression in a subgroup of HIV-1-positive women.

## Introduction

Women who are co-infected with Human Immunodeficiency Virus type 1 (HIV-1) and Human papillomavirus (HPV) are at high risk of developing cervical precancerous lesions (1). These precancerous lesions in HIV-1/HPV co-infected women are more aggressive, but only a small subset progress rapidly to invasive cervical cancer (ICC). This progression is unrelated to CD4 counts or antiretroviral (ARV) therapy (2, 3). What is not clear, however, is how and why this rapid cervical carcinogenesis is only observed in some HIV-1/HPV co-infected women (4).

Both HIV-1 and high-risk HPV (Hr-HPV) are classified as carcinogenic viruses (5). On the one hand, extrachromosomal HPV viral genomes often integrate into the host genome. This integration into the host genome drives the oncogenic process due to the overexpression of HPV viral oncoproteins E6 and E7 (6), which in turn, lead to inactivation of critical host DNA-damage-repair checkpoints during the cell cycle (7). Inactivation of the cell cycle checkpoints results in the accumulation of uncorrected mutations during normal DNA replication. These mutations increase host genomic instability and lead to further genetic alterations (8, 9). On the other hand, intracellular HIV-1 Tat proteins can interact directly with the *Rb* and *P53* tumor-suppressor genes in the host (10, 11). This interaction induces increased cell proliferation, which promotes the effects of HPV oncoproteins E6 and E7 in cervical carcinogenesis (12).

In two previous studies, we reported that, host molecular genetic variations at the Human Leucocyte Antigen class II (*HLA* II) locus on chromosome 6p and accumulation of repeated genetic alterations, can influence the rate of cervical disease progression in HIV-1/HPV co-infected women (4, 13). Furthermore, Harima et al. (14), reported that chromosome 6p was one of the chromosomal regions most frequently involved in the genetic alterations detected in cervical cancer. The availability of tumor biopsies in women with cervical disease can be used to interrogate the host genome for individualized tumor-specific early molecular oncogenic drivers (15). Loss of heterozygosity (LOH) and microsatellite instability (MSI) are among the most common earliest genetic alterations, and molecular oncogenic drivers, to occur in solid tumors including cervical cancer (14, 16). Both LOH and MSI may lead to the loss of microsatellite alleles, chromosomal loci, or single nucleotide polymorphisms (17).

MSI is a locus-specific change in the length of a short tandem repeat of nucleotide sequence in tumor genomic DNA when compared to the length in the normal genomic DNA (e.g., derived from white blood cells) from the same patient (18). MSI is caused by mutational inactivation of genes involved in DNA damage-repair (19). LOH at chromosomal level is the loss of one copy of an allele or a chromosomal locus in a certain region of a chromosome. If both copies of a gene are inactivated, LOH can result in inactivation of functional tumor-suppressor genes, oncogenes, immune-response genes, and DNA damage-repair genes that occur in the region of the chromosomal loss (20, 21). Inactivation of these important genes leads to physiologically uncontrolled cell growth and cell division in tissues where the LOH/MSI has occurred (22).

We previously hypothesized that HIV-1/HPV co-infection provokes additional genetic alterations at the *HLA* II locus to influence the rate of cervical disease progression in a subset of HIV-1-positive women (4). Furthermore, accumulation of repeated genetic alterations, can influence the rate of cervical disease progression in HIV-1/HPV co-infected women (4). In the early stages of the carcinogenic process, genetic alterations (LOH/MSI) can be identified in the tumor genomic DNA by using specific DNA markers (17). LOH/MSI can be studied in an individual's tumor genomic DNA, and compared to its status in a matched control genomic DNA from the same patient (17).

We investigated our hypothesis by using host genomic DNA fragments, analyzed in a multiplex polymerase chain reaction (PCR) using a capillary array electrophoresis platform. We used six fluorescently-labeled oligonucleotide primer pairs; BAT 26, D6S266, D6S2447, D6S1666, D6S2746, and D6S2881 to study chromosomal LOH/MSI in cervical tumor genomic DNA. These were compared to matched control DNA derived from buccal mucosa from the same patient, with regard to HIV-1/HPV co-infection in women histologically diagnosed with cervical disease in South Africa.

## Methods

### Research Ethics

All procedures were performed in accordance with guidelines of The Declaration of Helsinki. Ethical approval for the study was granted by the Human Research Ethics Committee of; the University of Cape Town (Number; HREC903/2015), all respective hospitals' Gynecology departments, the Department of Health of the Western Cape Government, and the South African National Health Laboratory Service. Consent forms were available in the language of the subject's choice and were signed in front of a witness. This was after detailed discussion with patients regarding the aims and nature of the study. A trained registered nurse who was fluent in the local languages explained the details of the study and answered all questions from the patients before their consent was requested.

### Study Design, Subjects, and Samples

As part of a large ongoing hospital-based project, a total of 200 patients were recruited from three hospitals in the Western Cape, namely; the Groote Schuur Hospital, Somerset Hospital and Victoria Wynberg Hospital between June 2016 to March 2017. All patients were referred from peripheral health centers to these three hospitals after receiving abnormal Pap smear results after routine cervical screening. Patients were recruited from: the outpatient gynecological cancer assessment clinics, colposcopy clinics, and the gynecological emergency rooms. The only criterion for recruitment was that the patient be newly diagnosed with cervical disease. The age distribution, in years, ranged from 24 to 91, with the majority of women in the age group between 30 and 40 years. Of these 200 women, 164 were fully investigated in this study.

Prior to the collection of buccal swabs, patients rinsed their mouths with sterile water, and mechanically chewed both inner buccal mucosal walls for at least 30 s. Both sides of buccal mucosa were scraped with a “DNA collector dry cotton swab stick” (Thermo Fisher Scientific, Johannesburg, South Africa) at least 20 times per side, as described according to the manufacturer's protocol. The buccal swabs were used to extract normal epithelial control DNA which were compared with abnormal tumor DNA from the same patients.

According to the South African HIV-1 testing algorithm, peripheral whole blood (4 ml) was collected in EDTA tubes (BD Vacutainer®, Johannesburg, South Africa). Approximately 20 μl of the collected peripheral whole blood was used for rapid HIV-1 antibody testing (Determine, Alere, Inc., Johannesburg, South Africa) (23).

Gynecologists used colposcopy inspection to collect punch biopsies of abnormal cervical lesions for histopathology analyses and HPV genotyping. All patients were recruited in this study before the initiation of the radiotherapy or chemotherapy treatment in order to avoid the consequences of DNA damage during cancer therapy (24).

### Tumor DNA Extraction and PCR Amplification

Genomic DNA was extracted using Qiagen® QIAamp DNA Mini purification kit (Qiagen, Johannesburg, South Africa) according to the manufacturer's protocol. The concentration of the extracted DNA was quantified by a Nanodrop® Spectrophotometer (Thermo Fisher Scientific, Johannesburg, South Africa). The DNA was diluted using nuclease free water (Thermo Fisher Scientific, Johannesburg, South Africa) to a recommended final concentration of 0.2 ng/μl of total DNA. The integrity of genomic DNA was tested by resolving DNA fragments on a 1% agarose gel by electrophoresis (Bio-Rad, Johannesburg, South Africa), migrated for 1 h at 100 V using 0.2 ng/μl of total DNA and 2 μL of orange loading dye (Thermo Fisher Scientific, Johannesburg, South Africa), followed by staining with ethidium bromide (Sigma-Aldrich, Johannesburg, South Africa) and visualization on a UV-transilluminator and the image was captured using a gel documentation system (Bio-Rad, Johannesburg, South Africa) (25). Each DNA sample was graded, according to the electrophoretic migration of sample DNA compared with 100 bp weight ladder (Thermo Fisher Scientific, Johannesburg, South Africa).

The extracted DNA was amplified by PCR-based assays using six fluorescently-labeled forward primers with the pair names, dyes and sequence as described in Table 1 (All primers were diluted to 20 ng/μl). The dream-Taq® PCR master-mix 12.5 μl (1.5 mmol MgCl2, 200 mmol dNTPs, 1 unit Taq DNA polymerase) (Thermo Fisher Scientific, Johannesburg, South Africa) was used for amplification according to the manufacturer's protocol. The PCR was carried out in 25 μl total reaction volumes, each containing 2 μl of template DNA, 1 μl of each primer (Forward and reverse) and 8.5 μl of nuclease free water (Thermo Fisher Scientific, Johannesburg, South Africa). The reaction mixture was heated to 95°C for 7 min, followed by 35 cycles, each consisting of 30 s denaturation at 94°C, 30 s annealing at 53°C, 30 s extension at 72°C, and a final 7-min extension at 72°C. The PCR amplification products (5 μl) were subjected to electrophoresis (Bio-Rad, Johannesburg, South Africa) on 1% agarose gel in 1 × Tris-acetate-EDTA buffer at 100 V for 1 h and stained with ethidium bromide (Sigma-Aldrich, Johannesburg, South Africa). The images were obtained in a gel documentation system (Bio-Rad, Johannesburg, South Africa) with the expected amplicon sizes for each marker as described in Table 1.

**Table 1:** Marker names, nucleotide sequences of primers, chromosomal positions, and the fluorescent dyes used for investigation of LOH and MSI at the respective loci.

**Marker**	**Primer sequence (5^**′**^ 3^**′**^)**	**Chromosomal region**	**Amplicon size (bp)**	**Motif**	**Fluorescent dye, 5^**′**^**
BAT26	F- TGACTACTTTTGACTTCAGCC	2p21	126	A(26)	FAM
	R- ACCCATTCAACATTTTTAACCC				
D6S2746	F-AGATTGTGCCACTGCACTCC	6p21.33	97–197	AAAAC	FAM
	R-ATAGTGCTGAGGTTGAGAGC				
D6S2881	F-GCTCGGGATTGAGAGGATTC	6p21.31	239–339	CA	PET
	R-AGCGGCGAGGTGAGCATGTC				
D6S266	F-GTTCCTCGGAATCATTTCCTCC	6p21.23	267–298	CTTT	FAM
	R-GGCAACAGAGTGAGGCTATCTTTG				
D6S1666	F-CTGAGTTGGGCAGCATTTG	6p21.32	131–151	GT	NED
	R-ACCCAGCATTTTGGAGTTG				
D6S2447	F-TTGAGAGGTGTGCATGTTAC	6p21.21	178–200	AC	VIC
	R-GCATTTCTCTTCCTTATCACTTC				

### HPV DNA Detection and Genotyping

Tumor genomic DNA was diluted using nuclease free water (Thermo Fisher Scientific, Johannesburg, South Africa) to reach a recommended final concentration of 0.2 ng/ μl of the total DNA. PCR-based Roche Linear Array® HPV genotyping test (Roche Molecular Systems, Pleasanton, CA, USA) which identifies 37 different HPV genotypes (HPV-6, -11, -16, -18, -26, -31, -33, -35, -39, -40, -42, -45, -51, -52, -53, -54, -55, -56, -58, -59, -61, -62, -64, -66, -67, -68, -69, -70, -71, -72, -73, -81, -82, -83, -84, -89 (HPV-CP6108) and –IS39) was used for typing HPV according to the manufacturer's instructions. All 37 different HPV genotypes were detected accordingly.

### Buccal Swab DNA Extraction and PCR Amplification

In order to preserve the DNA, all buccal swabs were air dried for at least 20 min after collection and then frozen at −20°C, until processed. The genomic DNA was extracted using Qiagen QIAamp DNA extraction kit (Qiagen, Johannesburg, South Africa) according to manufacturer's protocol (26). Briefly, each swab was placed in a 2 ml micro centrifuge tube and mixed with 20 μl proteinase K and 600 μl Buffer ATL, supplied in the kit. The mixture was placed in a thermomixer and incubated at 56°C with shaking at 900 rpm for at least 1 h; where after further extraction procedures were followed as per the manufacturer's protocol. All samples were eluted according to the manufacturer's instructions. The integrity of genomic DNA was tested as described in the previous section.

The extracted DNA was amplified by PCR using human beta-globin gene primers –Bg1 F(5′-CAACTTCATCCACGTTCACC-3′) and Bg2 R(5′-GAAGAGCCAAGGACAGGTAC-3′), and 12.5 μl Dream-Taq PCR master-mix (1.5 mmol MgCl2, 200 mmol dNTPs, 1 unit Taq DNA polymerase) (Thermo Fisher Scientific, Johannesburg, South Africa) according to the manufacturer's protocol. The PCR was carried out in 25 μl total reaction volumes, each containing 2 μl of template DNA, 0.5 μl of each primer, and 10.5 μl of nuclease free water (Thermo Fisher Scientific, Johannesburg, South Africa). The reaction mixture was heated to 95°C for 7 min, followed by 35 cycles, each consisting of 30 s denaturation at 94°C, 30 s annealing at 53°C, 30 s extension at 72°C, and a final 7-min extension at 72°C. The PCR amplification products (5 μl) were subjected to electrophoresis (Bio-Rad, South Africa) on 1% agarose gel in 1 × Tris-acetate-EDTA buffer at 100V for 1 h and stained with ethidium bromide (Sigma-Aldrich, Johannesburg, South Africa). The images were obtained in a gel documentation system (Bio-Rad, Johannesburg, South Africa) with expected amplicon size of 268 bp.

### LOH/MSI Analysis

LOH/MSI analysis was carried out in a “blinded” fashion, i.e., without knowledge of HIV-1 or HPV infection status. LOH/MSI was assessed at six highly polymorphic repeat markers; BAT26, D6S2447, D6S266, D6S2666, D6S2746, and D6S2881. The markers were chosen on the basis of their high heterozygosity informative content value of 0.7 in the *HLA* II gene and map position on chromosome 6p as described in the NCBI database (https://www.ncbi.nlm.nih.gov/) with supplementary mapping information, where necessary, provided in the Genome Reference Consortium (https://www.ncbi.nlm.nih.gov/grc) and the Human Genome Database (http://morissardjerome.free.fr/infobiogen/www.gdb.org/gdb/). The forward primers were labeled with fluorescent dyes: FAM, NED, PET, or VIC, as shown in Table 1 (27, 28).

Five of the markers studied are located in the *HLA* II locus (Chromosome 6p21), and one marker, BAT26, was located in the vicinity of the *MSH2* mismatch-repair gene on chromosome 2p21. BAT 26 marker was used in order to compare it with other markers in the analyses due to its high percentage of LOH and its reported accuracy in predicting LOH/MSI as previously studied in colorectal tumors (29).

Multiplex PCR reactions were performed and the amplified PCR products were analyzed by capillary array electrophoresis and GeneMapper® software (Applied Biosystems Inc., USA). After PCR, 0.5 μl of amplification product was mixed with 0.25 μl GS500-LIZ Size Standard and Hi-Di™ Formamide (Applied Biosystems, USA) to a final volume of 10 μl. The resulting mixture was denatured for 5 min at 95°C and then cooled on ice for at least 3 min. All the PCR products were genotyped on 3130 × 1 Genetic Analyser (Applied Biosystems Inc., Hitachi, USA), according to manufacturer's instructions.

**Figure 1:**
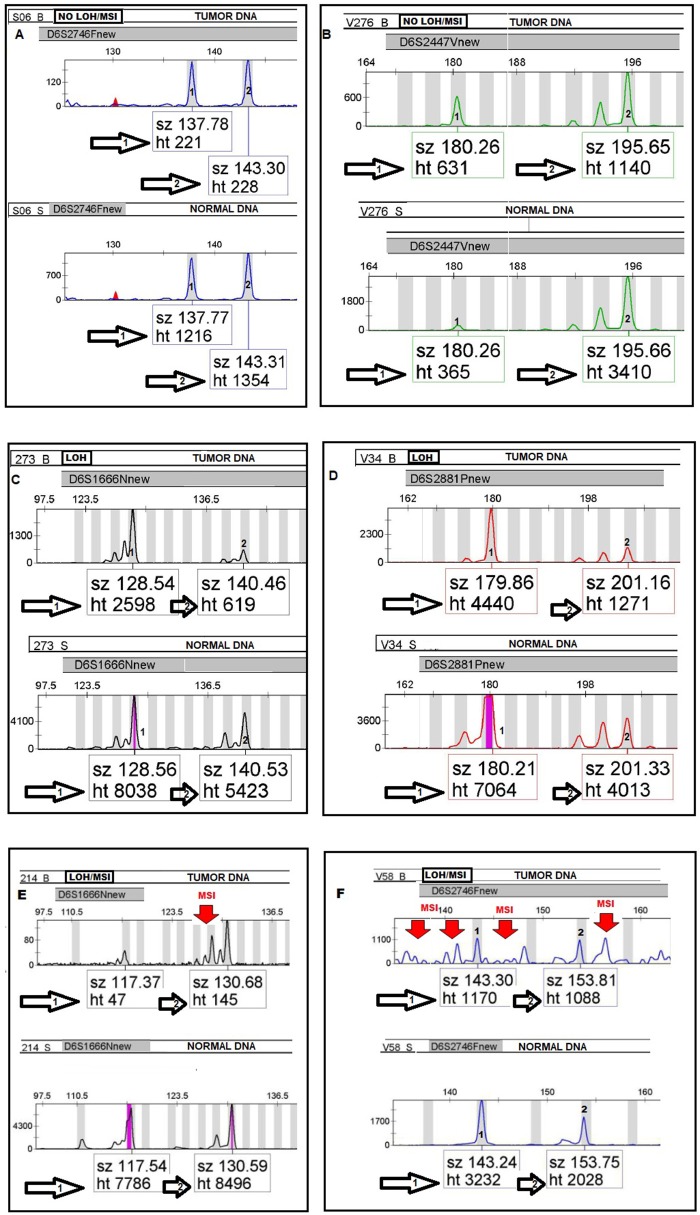
Electropherograms **(A–F)** illustrating the allelic status of tumor DNA compared to normal DNA in LOH/MSI analyses by using four fluorescently-labeled microsatellites markers. Paired tumor and normal heterozygous allele ratios were analyzed by allelic-imbalance factor by calculating the quotient of the peak ratios for each informative microsatellite alleles in the normal DNA divided by the corresponding ratio found in tumor DNA from the same patient as follows; Alleles in Normal 1:Normal 2/Tumor 1:Tumor 2.

MSI was defined as the presence of novel fragment sizes in DNA from tumor which was absent in the matched normal DNA from the same patient. Furthermore, for each informative tumor DNA/normal DNA pair, the allelic-imbalance ratio (AIR) was calculated. This is the ratio of the heights of both microsatellite alleles in the normal DNA divided by the ratio of heights in the tumor DNA from the same patient (17) (see Figure 1). An AIR value ≤ 0.67 or ≥ 1.35 was regarded as LOH (21). The LOH/MSI frequency was calculated as a percentage of LOH/MSI alterations present in relation to all informative loci (Heterozygous DNA) for each marker. At least two independent experiments were required to confirm the results in each event presenting with MSI/LOH.

### Statistical Analysis

Data analyses were based on previously published studies (30–32). The number of cases with LOH/MSI was divided by the total number of informative cases in that region to get the fractional locus loss. If one or more markers showed LOH/MSI, the locus was regarded as demonstrating LOH/MSI. Pearson chi-squared test for homogeneity of proportions using an exact *p-*value and multivariate logistic regression model were used for statistical analyses. LOH/MSI was used as a dependent variable for testing the significance of LOH/MSI variations between different predictor variables of interest within each marker. Normal distribution two-proportion Z-score test was used to test for a statistically significant difference between two proportions within the same categorical groups. We used sensitivity, specificity and area under the receiver operating characteristic (ROC) curves to assess if age was a predictor of ICC outcome in women with LOH/MSI. The *p-values* were corrected for false discovery rate (FDR) by the Benjamini-Hochberg test and the adjusted *p-values* (*q-values*) were reported. All odds ratios (ORs), 95% confidence intervals (95% CIs), the *p-values* and *q-values* calculated for multiple comparisons were 2-tailed, and considered significant if <0.05.

## Results

### Characteristics of the Study Cohort

In this study paired tumor (cervical lesions) and buccal swab DNA samples were available from 164 of the original 200 recruited patients. The paired samples were collected from 24 patients with mild cervical intraepithelial neoplasia (CIN1), 42 patients with moderate cervical intraepithelial neoplasia (CIN 2), 44 patients with severe cervical intraepithelial neoplasia (CIN 3 or carcinoma *in situ*). and 54 patients with ICC. Seventy-four patients were HIV-1-positive and ninety patients were HIV-1-seronegative. A total of 980 PCR reactions were performed, 490 each from matched tumor and buccal swab samples. The clinical and demographic features of the patients in the study cohort are summarized in Table 2. Six primer sets were used to analyse DNA microsatellites by a multiplex PCR, the number of paired samples examined for each marker is summarized in Table 3.

**Table 2:** Demographic features and the range of variables including clinical predictors measured in the subjects of the study cohort.

**Variable**	**BAT26 (*N* = 59)**	**D6S2881 (*N* = 81)**	**D6S2746 (*N* = 107)**	**D6S266 (*N* = 37)**	**D6S2666 (*N* = 97)**	**D6S2447 (*N* = 109)**
**Media*****n*** **age, y (Range)**	40 (26–77)	40 (26–77)	42 (28–77)	37 (24–61)	39 (24–66)	39 (24–91)
**Standard deviation**	9.7	9.7	9.6	8.4	8.7	10.6
**Age group (years)**	***n*** **(%)**	***n*** **(%)**	***n*** **(%)**	***n*** **(%)**	***n*** **(%)**	***n*** **(%)**
<30	1 (1.7)	4 (4.9)	5 (4.7)	2 (5.4)	6 (6.2)	7 (6.4)
30–40	30 (50.8)	39 (48.1)	46 (43)	21 (56.8)	54 (55.7)	60 (54.5)
> 40	28 (47.5)	38 (46.9)	56 (52.3)	14 (37.8)	37 (38.1)	42 (38.2)
**HIV−1 status**						
HIV−1–positive	36 (61)	46 (56.8)	56 (52.3)	19 (51.4)	62 (63.9)	62 (56.9)
HIV−1–seronegative	23 (39)	35 (43.2)	51 (47.7)	18 (48.6)	35 (36.1)	47 (43.1)
^*^HPV infection						
Single HPV infection	19 (32.2)	24 (29.6)	36 (33.6)	13 (35.1)	33 (34)	36 (33)
Multiple HPV infections	33 (55.9)	39 (48.1)	50 (46.7)	18 (48.6)	48 (49.5)	54 (49.5)
High risk HPV	46 (78)	59 (72.8)	81 (75.7)	27 (73)	77 (79.4)	86 (78.9)
Low risk HPV	6 (10.2)	4 (4.9)	5 (4.7)	4 (10.8)	4 (4.1)	4 (3.7)
**Tumor stage**						
CIN 1 & 2	20 (33.9)	31 (38.3)	38 (35.5)	17 (45.9)	44 (45.4)	49 (45)
CIN 3	16 (27.1)	17 (21)	25 (23.4)	9 (24.3)	25 (25.8)	29 (26.6)
Invasive	23 (39)	33 (40.7)	44 (41.1)	11 (29.7)	28 (28.9)	31 (28.4)
**Histopathology**						
Mild dysplasia	9 (15.3)	16 (19.8)	19 (17.8)	2 (5.4)	16 (16.5)	18 (16.5)
Moderate dysplasia	11(18.6)	15 (18.5)	19 (17.8)	15 (40.5)	28 (28.9)	31 (28.4)
Carcinoma *In situ*	16 (27.1)	17 (21)	25 (23.4)	9 (24.3)	26 (26.8)	29 (26.6)
Squamous cell carcinoma	20 (33.9)	26 (32.1)	35 (32.7)	8 (21.6)	23 (23.7)	24 (22)
Adenocarcinoma	3 (5.1)	7 (8.6)	9 (8.4)	3 (8.1)	4 (4.1)	7 (6.4)

* *14 Invalid HPV results were omitted in the analyses. CIN 1, cervical intraepithelial neoplasia 1(Mild); CIN 2, cervical intraepithelial neoplasia 2 (Moderate); CIN 3, cervical intraepithelial neoplasia 3 (Severe or carcinoma in situ)*.

**Table 3:** Number of paired samples for which each microsatellite marker was successfully resolved.

	**Marker**	**Samples**
i)	BAT26	59 paired samples
ii)	D6S2881	81 paired samples
iii)	D6S2746	107 paired samples
iv)	D6S266	37 paired samples
v)	D6S2666	97 paired samples
vi)	D6S2447	109 paired samples

#### Comparisons of LOH/MSI Frequency

- **Between tumor DNA from precancerous lesions and ICC**
  To investigate whether LOH/MSI was different between patients with precancerous lesions and ICC, the frequency of LOH/MSI in tumor DNA from precancerous lesions and ICC for each marker was compared separately. ICC tumor DNA showed more LOH/MSI only at 6p21.31 (D6S2881) than precancerous lesions tumor DNA, i.e., 78.8 vs. 52.1%, respectively, *p* = 0.019. However, the false discovery rate (FDR) q-value was not statistically significant (*q* = 0.114; Table 4A).
- **Between tumor DNA from HIV-1-positive and HIV-1-seronegative women**
  To investigate if LOH/MSI was different between tumor biopsies, depending on HIV-1 infection status, the frequency of LOH/MSI for all tumor biopsy DNA was compared separately in HIV-1-positive and HIV-1-seronegative women. The results show that tumor DNA from HIV-1-positive women demonstrated a higher frequency of LOH/MSI than tumor DNA from HIV-1-seronegative women at 6p21.21 (D6S2447, 74.2 vs. 42.6%; *p* = 0.001, *q* = 0.003), 6p21.31 (D6S2881, 78.3 vs. 42.9%; *p* = 0.002, *q* = 0.004), 6p21.32 (D6S2666, 79 vs. 57.1%; *p* = 0.035, *q* = 0.052), and 6p21.33 (D6S2746, 64.3 vs. 29.4%; *p* < 0.001, *q* < 0.001), respectively (Table 4B).

**Table 4:** Frequency of LOH/MSI according to cervical disease and HIV-1 status.

**A**						**B**						
	**Precancerous**		**Invasive cancer**				**HIV-1-seronegative**		**HIV-1-positive**			
**Marker**	**Cases studied**	**LOH/MSI (%)**	**Cases studied**	**LOH/MSI (%)**	***p*****-value**	**FDR** ***q*****-value**	**Cases studied**	**LOH/MSI (%)**	**Cases studied**	**LOH/MSI (%)**	***p*****-value**	**FDR** ***q*****-value**
BAT 26	36/59	6/36 (16.7)	23/59	6/23 (26.1)	0.51	1.02	23/59	3/23 (13)	36/59	9/36 (25)	0.334	0.401
D6S266	26/37	12/26 (46.2)	11/37	4/11 (36.4)	0.723	1.085	18/37	9/18 (50)	19/37	7/19 (36.8)	0.515	0.515
D6S2666	69/97	49/69 (71)	28/97	20/28 (71.4)	>0.99	0.99	35/97	20/35 (57.1)	62/97	49/62 (79)	**0.035**	0.052
D6S2881	48/81	25/48 (52.1)	33/81	26/33 (78.8)	**0.019**	0.114	35/81	15/35 (42.9)	46/81	36/46 (78.3)	**0.002**	**0.004**
D6S2746	63/107	33/63 (52.4)	44/107	18/44 (40.9)	0.325	0.975	51/107	15/51 (29.4)	56/107	36/56 (64.3)	** <0.001**	** <0.001**
D6S2447	78/109	48/78 (61.5)	31/109	18/31 (58.1)	0.829	0.99	47/109	20/47(42.6)	62/109	46/62 (74.2)	**0.001**	**0.003**

***(A)** Frequency of LOH/MSI between precancerous lesions and ICC. **(B)** Frequency of LOH/MSI between HIV-1-seronegative and HIV-1-positive women (Pearson chi-square test for homogeneity of proportions by using an exact p-value). Bold values show significant p-values*.

The present study also compared the odds of having LOH/MSI (LOH/MSI-positive vs. LOH/MSI-negative cases) according to HIV-1 status for each marker. The results show that tumor biopsy DNA from HIV-1-positive women had a higher relative risk of having LOH/MSI than tumor biopsy DNA from HIV-1-seronegative women for markers; D6S2881 (OR 4.8, CI 1.8–12.7, *p* = 0.002, *q* = 0.004), D6S2746 (OR 4.3, CI 1.9–9.7, *p* < 0.001, *q* = <0.001), D6S2666 (OR 2.8, CI 1.1–7.7, *p* = 0.035, *q* = 0.052), and D6S2447 (OR 3.88, CI 1.6–9.5, *p* = 0.001, *q* = 0.003; Table 5).

**Table 5:** The relationship between the odds of having LOH/MSI and HIV-1 status for each marker.

**Outcome**						
	**BAT 26 (*****n*** **= 59)**					
**Exposure**	**No LOH/MSI (*****n*** **= 47)**	**LOH/MSI (*****n*** **= 12)**	**OR**	**CI**	***p*****-value**	**FDR** ***q*****-value**
HIV-1-seronegative (*n* = 23)	20 (42.6%)	3 (25%)	Ref.			
HIV-1-positive (*n* = 36)	27 (57.4%)	9 (75%)	2.2	0.5–14	0.334	0.401
	**D6S2881 (*****n*** **= 81)**					
	**No LOH/MSI (*****n*** **= 30)**	**LOH/MSI (*****n*** **= 51)**				
HIV-1-seronegative (*n* = 35)	20 (66.7%)	15 (29.4%)	Ref.			
HIV-1-positive (*n* = 46)	10 (33.3%)	36 (70.6%)	4.8	1.8–12.7	**0.002**	**0.004**
	**D6S2746 (*****n*** **= 107)**					
	**No LOH/MSI (*****n*** **= 56)**	**LOH/MSI (*****n*** **= 51)**				
HIV-1-seronegative (*n* = 51)	36 (64.3%)	15 (29.4%)	Ref.			
HIV-1-positive (*n* = 56)	20 (35.7%)	36 (70.6%)	4.3	1.9-9.7	** <0.001**	** <0.001**
	**D6S266 (*****n*** **= 37)**					
	**No LOH/MSI (*****n*** **= 21)**	**LOH/MSI (*****n*** **= 16)**				
HIV-1-seronegative (*n* = 18)	9 (42.9%)	9 (56.2%)	Ref.			
HIV-1-positive (*n* = 19)	12(57.1%)	7 (43.8%)	0.58	0.1–2.6	0.515	0.515
	**D6S2666 (*****n*** **= 97)**					
	**No LOH/MSI (*****n*** **= 28)**	**LOH/MSI (*****n*** **= 69)**				
HIV-1-seronegative (*n* = 35)	15 (53.6%)	20 (29%)	Ref.			
HIV-1-positive (*n* = 62)	13 (46.4%)	49(71%)	2.8	1.1–7.7	**0.035**	0.052
	**D6S2447 (*****n*** **= 109)**					
	**No LOH/MSI (*****n*** **= 43)**	**LOH/MSI (n= 66)**				
HIV-1-seronegative (*n* = 47)	27 (62.8%)	20 (30.3%)	Ref.			
HIV-1-positive (*n* = 62)	16 (37.2%)	46 (69.7%)	3.88	1.6–9.5	**0.001**	**0.003**

*Bold values show significant p-values*.

### Comparison Between LOH/MSI Status and Clinical Variables

In order to control for the effects of other variables that may influence the outcome, a multivariate logistic regression model was used to study the association of each variable by considering LOH/MSI as a dependent variable for four markers that showed significantly different frequency among the studied groups. In this analysis, only HIV-1 status was significantly associated with LOH/MSI in DNA marker; D6S2746 (*p* < 0.0001, *q* < 0.001), D6S2881 (*p* = 0.025, *q* = 0.15), D6S2447 (*p* = 0.002, *q* = 0.012), and D6S2666 (*p* = 0.021, *q* = 0.063) while tumor stage and histopathology were significantly associated with LOH/MSI in DNA marker D6S2666 for tumor stage (*p* = 0.027, *q* = 0.054) and for histopathology (*p* = 0.015, *q* = 0.09; Table 6). Furthermore, aging and cancer are highly interconnected, older age being a significant risk factor for cancer development (33). However, in cervical cancer, it has already been reported that HIV-1-positive women develop ICC earlier and at a younger age compared to HIV-1-seronegative women (34–36). Since there is limited data on the impact of age on LOH/MSI in cervical cancer development amongst HIV-1-positive women, this investigation sought to study whether age could predict ICC outcome in HIV-1-positive women with LOH/MSI in the study cohort by plotting specificity against sensitivity for each marker by using (ROC) curves to calculate the area under the curves (AUC), with *p*-values. This study found that age could predict ICC outcome in HIV-1-positive women with LOH/MSI for two DNA markers, D6S2447 (*p* = 0.0224, *q* = 0.044) and D6S2666 (*p* = 0.01, *q* = 0.02; Supplementary Figure 1).

**Table 6:** Multivariate analysis in logistic regression for each marker by using LOH/MSI as a dependent variable.

	**D6S2746 (N = 107)**	***p*-value**	**FDR *q*-value**	**D6S2881 (N = 81)**	***p*-value**	**FDR *q*-value**	**D6S2447 (N = 109)**	***p*-value**	**FDR *q*-value**	**D6S2666 (N = 97)**	***p*-value**	**FDR *q*-value**
**Variables**												
**Age (in years)**	n (%)	0.256	0.384	*n* (%)	0.687	1.031	*n* (%)	0.16	0.48	*n* (%)	0.7	0.84
<30	5 (4.7)			4 (4.9)			7 (6.4)			6 (6.2)		
30-40	46 (43)			39 (48.1)			60 (55)			54 (55.7)		
>40	56 (52.3)			38 (46.9)			42 (38.5)			35 (36.1)		
**HIV-1 status**		** <0.0001**	** <0.001**		**0.025**	0.15		**0.002**	**0.012**		**0.021**	0.063
HIV-1-seronegative	51 (47.7)			35 (43.2)			62 (56.9)			35 (36.1)		
HIV-1-positive	56 (52.3)			46 (56.8)			47 (43.1)			62 (63.9)		
**HPV risk**		0.174	0.522		0.837	1.004		0.997	0.997		0.919	0.919
Low risk HPV	5 (4.7)			4 (4.9)			4 (3.7)			4 (4.1)		
High risk HPV	81 (75.7)			59 (72.8)			86 (78.9)			77 (79.4)		
**HPV single or multiple**		0.215	0.43		0.935	0.935		0.353	0.706		0.142	0.213
Single HPV	36 (33.6)			24 (29.6)			36 (33)			33 (34)		
Multiple HPVs	50 (46.7)			39 (48.1)			54 (49.5)			48 (49.5)		
**Tumor stage**		1.171	1.171		0.632	1.264		0.818	0.981		**0.027**	0.054
CIN 1 & 2	38 (35.5)			31 (38.3)			49 (45)			44 (45.4)		
CIN 3	25 (23.4)			17 (21)			29 (26.6)			25 (25.8)		
Invasive	44 (41.1)			33 (40.7)			31 (28.4)			28 (28.9)		
**Histopathology**		0.646	0.775		0.267	0.801		0.536	0.804		**0.015**	0.09
Mild dysplasia	19 (17.8)			16 (19.8)			18 (16.5)			16 (16.5)		
Moderate dysplasia	19 (17.8)			15 (18.5)			31 (28.4)			28 (28.9)		
Carcinoma *in situ*	25 (23.4)			17 (21)			29 (26.6)			26 (26.8)		
SCC	35 (32.7)			26 (32.1)			24 (22)			23 (23.7)		
ADC	9 (2.8)			7 (8.6)			7 (6.4)			4 (4.1)		

*Bold values show significant p-values. SCC, squamous cell carcinoma; ADC, Adenocarcinoma*.

### Comparison of LOH/MSI Status Between Tumor DNA From Precancerous Lesions and ICC According to HIV-1 Infection

**Figure 2:**
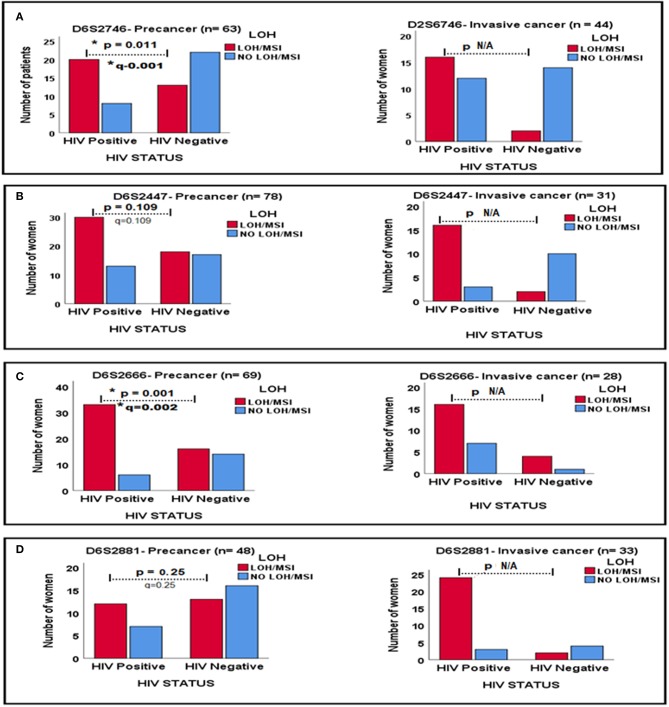
Relationships between LOH/MSI status and HIV-1 status between precancerous lesions and ICC biopsies for four DNA markers in **(A–D)**. ^*^Signs mean significant *p*-value and significant *q*-value, respectively.

In order to further examine the effects of HIV-1 infection on the status of LOH/MSI, a comparison was made of LOH/MSI between tumor DNA from precancerous lesions and ICC according to HIV-1 infection for four markers; In marker D6S2746, tumor DNA from HIV-1-positive women showed significantly more LOH/MSI than tumor DNA from HIV-1-seronegative women with precancer (*p* = 0.011, *q* = 0.011). For marker D6S2447, tumor DNA from HIV-1-positive women showed more LOH/MSI than tumor DNA from HIV-1-seronegative women in invasive cancer, although the *p-value* was not applicable (N/A) due to a very low number of HIV-1-seronegative women with LOH/MSI. For marker D6S2666, tumor DNA from HIV-1-positive women showed significantly more LOH/MSI than tumor DNA from HIV-1-seronegative women with precancer (*p* = 0.001, *q* = 0.002), the *p-value* was not applicable for invasive cancer due to a very low number of HIV-1-seronegative women with LOH/MSI. Finally, for marker D6S2881, tumor DNA from HIV-1-positive women showed more LOH/MSI than tumor DNA from HIV-1-seronegative women with ICC, although the *p-value* was not applicable due to very low number of HIV-1-seronegative women with LOH/MSI (*p* = 0.011, *q* = 0.022; Figure 2).

### Comparison of LOH/MSI Status Between HIV-1-Positive and HIV-1-Seronegative Women With Hr-HPV Infection

**Figure 3:**
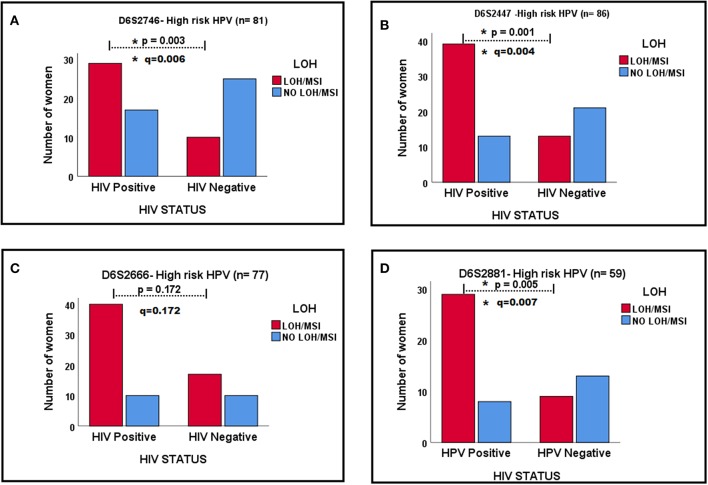
The relationships in LOH/MSI status between HIV-1-positive and HIV-1-seronegative women with Hr-HPV infection in four markers in **(A–D)**. ^*^Signs mean significant *p*-value and significant *q*-value, respectively.

Because Hr-HPV infection is a known risk factor for cervical disease development, and HIV-1 infection increases prevalence, persistence and reduces clearance of HPV-infection (1), this study examined whether LOH/MSI between tumor (cervical lesion) biopsy DNA from HIV-1-positive women and HIV-1-seronegative women differ according to Hr-HPV infection in four markers which showed significant results. Tumor biopsy DNA from HIV-1-positive women with Hr-HPV infection showed more LOH/MSI than tumor biopsy DNA from HIV-1-seronegative women with Hr-HPV infection in marker D6S2746 (*p* = 0.003, *q* = 0.006; Figure 3A), D6S2447 (*p* = 0.001, *q* = 0.004; Figure 3B) and D6S2881 (*p* = 0.005, *q* = 0.007; Figure 3D). However, with marker D6S2666, the difference was not statistically significant *p* = 0.172, *q* = 0.172 (Figure 3C).

## Discussion

The present study represents the first in-depth analysis of cancerous changes, viral infection, and host LOH/MSI. The study performed comprehensive allelo-typing of isolated genomic DNA from buccal swab samples (control DNA), and from cervical precancerous lesions and ICC samples. All these samples were from HIV-1-positive and HIV-1-seronegative South African women, none of them were HPV vaccinated before. More importantly, this study is the first to offer an opportunity to examine the effects of HIV-1/HPV co-infection in cervical cancer by using a host molecular genetics approach. The findings revealed a significantly higher frequency of chromosomal LOH/MSI in tumor biopsy DNA from HIV-1-positive women than from HIV-1-seronegative women at the *HLA* II locus on chromosome 6p, as summarized in Supplementary Table 1. The investigation also contributes some important information to the existing theories of host molecular genetic alterations and cervical carcinogenesis in HIV-1-positive women that requires further investigation.

Although the duration of oncogenic HPV infection is recognized as an etiologic factor for the development of cervical disease, other host factors, and an understanding of the mechanisms involved in the cervical carcinogenesis pathway in the host, remain unknown (37). In view of the association of certain *HLA* II genes on chromosome 6p with cervical cancer, this study has considered the molecular genetic basis of this association in the host. The present study reports LOH/MSI from tumor DNA in precancerous lesions and cancerous lesions even from HIV-1-seronegative women. These results suggest that, in HPV infected women with cervical disease, LOH/MSI is an early genetic event in the development of cervical cancer, including the pre-invasive lesions. HIV-1 infection is an additional factor that appears to change the genetic makeup of the host cell, which in turn causes the overall genomic instability of cervical tumor DNA during the carcinogenic process. Although HPV infection alone can induce LOH/MSI at the *HLA* II locus in cervical tumor DNA, HIV-1 co-infection exacerbates it, potentially accelerating cervical disease progression in a subgroup of HIV-1-positive women.

Since LOH/MSI play an important role in cervical carcinogenesis (21), five locus-specific DNA markers were used to study LOH/MSI at the *HLA* II locus on chromosome 6p. This region contains the *HLA* II, specifically, -DRB1 and -DQB1 which are responsible for viral infection recognition and antigen presentation to the immune system (38). Loss of an allele, or loss of a part of a chromosome at the *HLA* II locus, may lead to haplo-insufficiency, and can have multiple functional effects on viral immune response genes. Furthermore, if one allele of a tumor-suppressor gene is inactivated earlier at a specific locus by somatic mutations, then deletion of a second allele, as detected by LOH, may result in loss of function of a tumor-suppressor gene or an immune-response gene (39). These results strongly support the argument that the *HLA* II locus on chromosome 6p is critical in the pathogenesis of cervical cancer. The present study has demonstrated that several genetic alterations associated with different cervical disease stages regardless of HIV-1 infection status. Findings similar to these have been reported by Harima et al. in Japan (14), by Mazurenko et al. in Russia (40, 41) and by Pulido et al. in the USA (30). However, the specific tumor-suppressor gene(s) at this locus remain unknown in different study populations.

In a previous study (13), we described an association, or likely protection from cervical cancer in HIV-1/HPV co-infected South African women, with certain *HLA* II genotypes. Furthermore, a study carried out by Meys et al. (42) reported that specific *HLA* immunogenotypes can determine the persistence of HPV infection in HIV-1 infected patients even during antiretroviral treatment. The presence of HIV-1/HPV co-infection in combination with specific *HLA* II genotypes or a haplotype may increase or decrease the risk of cervical disease development. Therefore, according to the present study, in the case of likely protective *HLA* II (*HLA* II alleles associated with decreased cancer risk), presence of LOH/MSI at the *HLA* II locus may further affect the protective function of these genes on HPV infection clearance (43). Additionally, genomic changes and genetic alterations in the cervical pre-cancerous lesions and ICC may be induced differently by different types of HPV, and particular HPV-specific *HLA* II acting in combination (44–46). Since cervical cancer is a complex genetic disease, this thesis acknowledges that the influence of epigenetics and other genomic changes on cervical cancer progression (47, 48), may also play a part in this process.

The presence of MSI is phenotypic evidence that DNA mismatch repair is not functioning normally (49). Therefore, structural genomic changes within the *HLA* II locus, may determine cervical lesions that are likely to progress to invasive cancer due to inactivation of immune-response genes and the persistence of oncogenic HPV infection. HIV-1 Tat proteins can also interact directly with functional tumor-suppressor genes in the host (*Rb* and *p53*) (10, 11), which induces increased cell proliferation and increases the effect of Hr-HPV oncoproteins E6 and E7 in cervical carcinogenesis (4, 12).

Genomic instability is an early event in carcinogenesis and arises as a consequence of the disruption of critical cell-cycle check-points and failure of the DNA damage-repair system as observed in premalignant tumor DNA (Table 4 and Figure 2). These findings are similar to those of Migdalska-Sęk et al. (21) who reported LOH/MSI as early events in precancerous lesions according to HPV infection status, however, the HIV-1 status of their patients was unknown. Presence of LOH/MSI in premalignant tumors allows cells to acquire the additional mutations, required for malignant transformation (50). The present study further suggests that genetic instabilities are early trigger genetic events which may facilitate the subsequent establishment of all other hallmarks of cancer.

Other studies performed on LOH/MSI in head and neck cancers, colon cancer, ovarian cancer, and in cervical cancer in different populations with unknown HIV-1 status, reported frequent LOH/MSI in many chromosomes, with LOH/MSI frequencies that varied from 17 to 90% (9, 17, 18, 21, 30, 51). However, in this study, the LOH/MSI frequencies vary from 13 to 79% (Table 5). The discrepancy between the present study and other published works could be due to;

- The quality and concentration of genomic DNA.
- Differences in the specific DNA markers used.
- Intrinsic genetic differences in genomic DNA composition among different populations.
- The specific cancer disease of interest, whether it is MSI-High, MSI-Low, microsatellite stable or unstable (52).
- The particular chromosomes examined.
- The specific PCR optimization conditions used (53).

The presence of dual-oncogenic HPV and HIV-1 infections has remarkable effects on genomic instability in our study population. This was demonstrated by the observation that Hr-HPV infection influenced the frequency of LOH/MSI at the *HLA* II locus more in cervical tumor DNA from HIV-1-positive women than cervical tumor DNA from HIV-1-seronegative women, except in DNA marker D6S2666 (*p* = 0.172; Figure 3C). However, when this study examined the odds of having LOH/MSI with other predictor variables in a multivariate logistic regression analysis, LOH/MSI was significantly associated only with HIV-1 status, in all four markers (Tables 5, 6). This may be due to the effects of including many predictor variables at once, which can dilute the true association, scientific plausibility, and clinical meaningfulness of any individual result (32). However, the present study obtained interesting results by using the ROC curves (Supplementary Figure 1) that age was a significant predictor of ICC outcome in HIV-1-positive women with LOH/MSI in DNA markers D6S2447 and D6S2666. In combination with Tables 5, 6, these results suggest that, age above 30 years is a strong poor prognostic factor for ICC in HIV-1-positive women with LOH/MSI by using these two markers. Further studies are warranted on the differential effects of specific HPV genotypes and HIV-1 co-infection on the overall genomic instability of cervical tumor DNA during carcinogenesis.

The unique strengths of this study include the comparative molecular investigation of the frequency of LOH/MSI between tumors from HIV-1-positive and HIV-1-seronegative women. The examination of abnormal cervical tumor epithelial DNA and matched normal buccal mucosa epithelial DNA, as control DNA. Previously published studies have focused almost exclusively on control DNA from whole blood, which may not provide a reasonable comparison with a matched abnormal epithelial tumor DNA. Previously published studies examining tumor biopsies, have not reported on cervical tumors from HIV-1-positive women. The present analysis has focused on chromosome 6p21 only, with five different locus-specific DNA markers to amplify fractions of the *HLA* II locus. Mononucleotide repeats were disregarded due to difficult marker analyses and difficulty in distinguishing heterozygotes from homozygotes if the allele sizes were very similar, because of “stutters.” The capillary electrophoresis analyses were repeated by using ethanol precipitation to clean the PCR products, wash out excess salts and unincorporated primer leftover after PCR for samples which showed poor amplification. PCR-based LOH assays include the ability to detect small deletions and the ability to enrich for tumor cells through microdissection (54). This investigation has been able to answer the research question by demonstrating that HIV-1/HPV co-infection does provoke additional LOH/MSI in cervical tumor DNA which may influence the rate of cervical disease progression in a subgroup of HIV-1-positive women.

The study limitations include the possibility of contaminated tumor samples since histologic sections of tumors usually contain a mixture of tumor cells, inflammatory cells, stromal cells, and other cellular contaminants. Limited molecular data from the study population on the presence of polymorphisms at the primer binding sites, therefore null alleles could not be excluded. Relative fluorescent units (RFUs) were very high for some swab samples and very low or absent for others which reflects unequal amplification between swab samples. In case of inadequate DNA concentration or low DNA concentration, one allele may be preferentially amplified over the other, and where one allele has insufficient amplification, termed *allelic dropout*. Off-scale peaks could not be sized accurately and it was not possible to determine the peak heights when the camera was saturated above 8,000 RFUs. Finally, we did not make any patient follow up in our study.

Future research opportunities may include *in vivo* studies to demonstrate the mechanisms of HIV-1/HPV co-infection in cervical carcinogenesis with specific *HLA* II alleles or haplotypes combinations, and genome-wide assays including massively parallel DNA sequencing and single-nucleotide polymorphism arrays to investigate further in-depth mechanisms and the presence of cause-effect relationships of our findings.

## Conclusions

This study has demonstrated a unique relationship between LOH/MSI in cervical tumor DNA and HIV-1/HPV co-infection in a cohort of South African women. Tumor DNA from HIV-1/HPV co-infected women demonstrated a higher frequency of LOH/MSI than tumor DNA from HIV-1-seronegative women at chromosome 6p. Loss of an allele or part of a chromosome can have multiple functional effects on immune-response genes, DNA damage-repair genes and tumor-suppressor genes. The results suggest that HPV infection alone can induce LOH/MSI at the *HLA* II locus in cervical tumor DNA, whereas HIV-1 co-infection exacerbates it, possibly accelerating cervical disease progression in a subgroup of HIV-1-positive women. This work adds to the existing theories of host molecular genetic alterations and cervical carcinogenesis.

## Data Availability Statement

The raw data supporting the conclusions of this manuscript will be made available by the authors, without undue reservation, to any qualified researcher.

## Ethics Statement

The studies involving human participants were reviewed and approved by all procedures performed in this study involving human participants were in accordance with the ethical standards of the institutional and/or national research committee and with the 1964 Helsinki declaration and its later amendments or comparable ethical standards. The patients/participants provided their written informed consent to participate in this study.

## Author Contributions

RC and RR conceived of the presented idea and developed the theory. LD and others collected the specimens. EK and GA contributed in the laboratory works of the research. Together RR, GR, A-LW, MM-S, and CG encouraged RC to investigate further, to carry out specific experiments and supervised final results and findings of this work. RC performed the computations and verified the statistical analyses methods. All other authors discussed the results and contributed equally to the final preparations of the manuscript.

### Conflict of Interest

Opinions, findings and conclusions, or recommendations expressed in this publication generated by the listed organizations supported research is that of the authors alone, and that the organizations accept no liability whatsoever in this regard. The authors declare that the research was conducted in the absence of any commercial or financial relationships that could be construed as a potential conflict of interest.

## Supplementary Material

Supplementary Figure 1 Receiver operating characteristic curves by using age of patients to predict ICC between HIV-1-positive and HIV-1-seronegative women with LOH/MSI for four significant DNA markers. **(A)** ROC curves for DNA marker D6S2746, showing prediction for ICC with LOH/MSI in both HIV-1-positive and HIV-1-seronegative women by using age. **(B)** ROC curves for DNA marker D6S2447, showing prediction for ICC with LOH/MSI in both HIV-1-positive and HIV-1-seronegative women by using age. **(C)** ROC curves for DNA marker D6S2666, showing prediction for ICC with LOH/MSI in both HIV-1-positive and HIV-1-seronegative women by using age. **(D)** ROC curves for DNA marker D6S2881, showing prediction for ICC with LOH/MSI in both HIV-1-positive and HIV-1-seronegative women by using age.

Supplementary Table 1 Summary of the results. Where; AUC, Area under the curve.
