# Supplementary figures and images for: Investigation of Cervical Tumor Biopsies for Chromosomal Loss of Heterozygosity (LOH) and Microsatellite Instability (MSI) at the HLA II Locus in HIV-1/HPV Co-infected Women
Source: Front Oncol. 2019 Oct 15;9:951. doi: 10.3389/fonc.2019.00951 (PMC6803484; doi:10.3389/fonc.2019.00951)

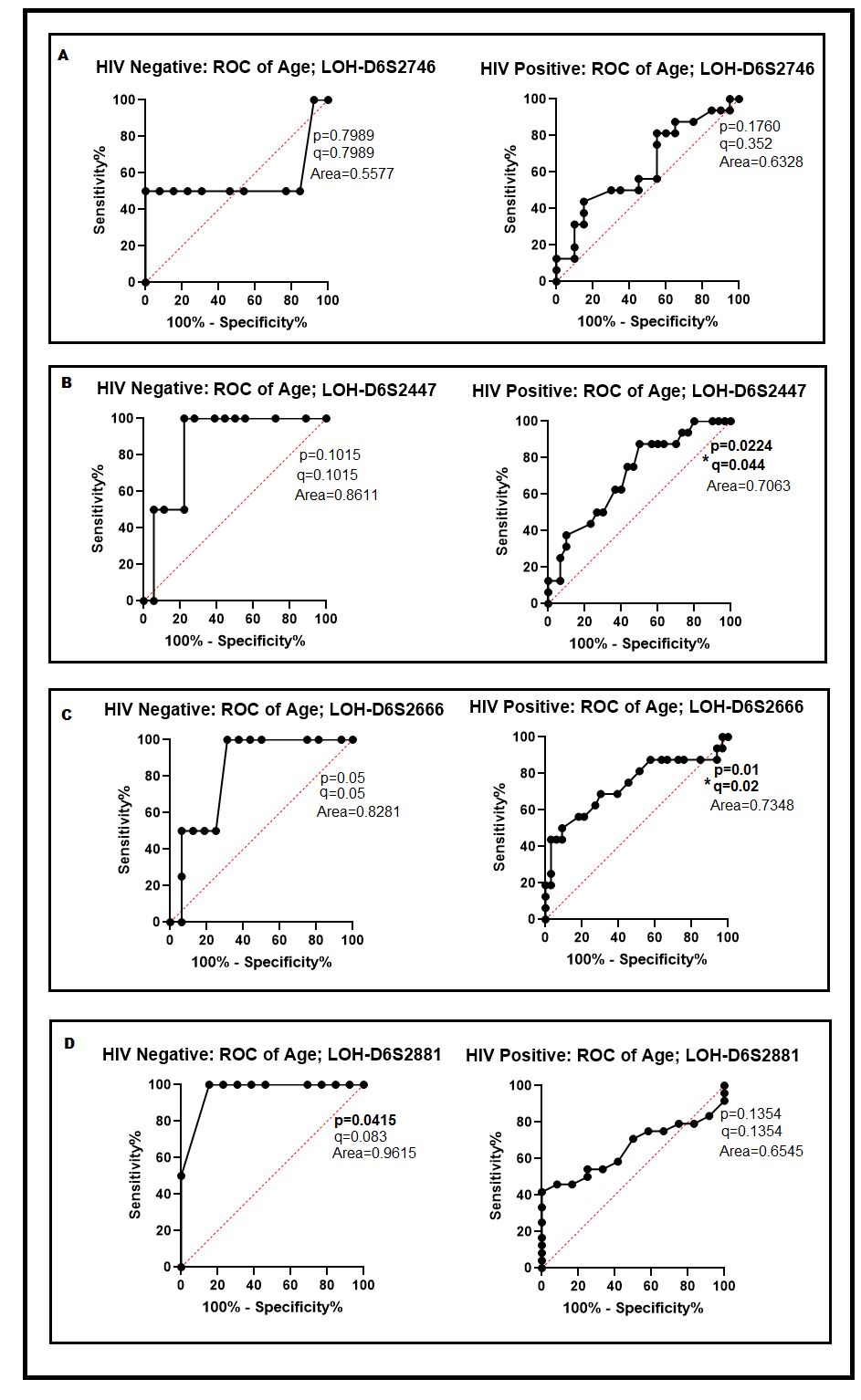

Supplement: Supplementary Figure 1 — Receiver operating characteristic curves by using age of patients to predict ICC between HIV-1-positive and HIV-1-seronegative women with LOH/MSI for four significant DNA markers. (A) ROC curves for DNA marker D6S2746, showing prediction for ICC with LOH/MSI in both HIV-1-positive and HIV-1-seronegative women by using age. (B) ROC curves for DNA marker D6S2447, showing prediction for ICC with LOH/MSI in both HIV-1-positive and HIV-1-seronegative women by using age. (C) ROC curves for DNA marker D6S2666, showing prediction for ICC with LOH/MSI in both HIV-1-positive and HIV-1-seronegative women by using age. (D) ROC curves for DNA marker D6S2881, showing prediction for ICC with LOH/MSI in both HIV-1-positive and HIV-1-seronegative women by using age. [file Image_1.PNG]
