# Supplementary material for: Investigation of Cervical Tumor Biopsies for Chromosomal Loss of Heterozygosity (LOH) and Microsatellite Instability (MSI) at the HLA II Locus in HIV-1/HPV Co-infected Women
Source: Front Oncol. 2019 Oct 15;9:951. doi: 10.3389/fonc.2019.00951 (PMC6803484; doi:10.3389/fonc.2019.00951)
Supplement: Supplementary Table 1 — Summary of the results. Where; AUC, Area under the curve. [file Table_1.docx]

**Supplementary Table 1**. Summary of the results

|  | **LOH/MSI, n/N (%)** | **LOH/MSI, n/N (%)** | | | ***p-value*** | ***q-value*** |
| --- | --- | --- | --- | --- | --- | --- |
|  | **Cervical disease stage** |  | | |  |  |
| **DNA marker** | **Pre-invasive lesions** | **Invasive cancer** | | |  |  |
| BAT 26 | 6/36 (16.7) | 6/23 (26.1) | | | 0.51 | 1.02 |
| D6S266 | 12/26 (46.2) | 4/11 (36.4) | | | 0.723 | 1.085 |
| D6S2666 | 49/69 (71) | 20/28 (71.4) | | | >0.99 | 0.99 |
| D6S2881 | 25/48 (52.1) | 26/33 (78.8) | | | **0.019** | 0.114 |
| D6S2746 | 33/63 (52.4) | 18/44 (40.9) | | | 0.325 | 0.975 |
| D6S2447 | 48/78 (61.5) | 18/31 (58.1) | | | 0.829 | 0.99 |
|  |  |  | | |  |  |
|  | **HIV-1 status** |  | | |  |  |
| **DNA marker** | HIV-1-seronegative | HIV-1-positive | | |  |  |
| BAT 26 | 3/23 (13) | 9/36 (25) | | | 0.334 | 0.401 |
| D6S266 | 9/18 (50) | 7/19 (36.8) | | | 0.515 | 0.515 |
| D6S2666 | 20/35 (57.1) | 49/62 (79) | | | **0.035** | 0.052 |
| D6S2881 | 15/35 (42.9) | 36/46 (78.3) | | | **0.002** | **0.004** |
| D6S2746 | 15/51 (29.4) | 36/56 (64.3) | | | **<0.001** | **<0.001** |
| D6S2447 | 20/47(42.6) | 46/62 (74.2) | | | **0.001** | 0.003 |
|  |  |  | | |  |  |
|  | **High risk HPV** |  | | |  |  |
| **DNA marker** | HIV-1-seronegative | HIV-1-positive | | |  |  |
| D6S2666 | 18/77 (23.4) | 40/77 (51.9) | | | 0.172 | 0.172 |
| D6S2881 | 10/59 (16.9) | 30/59 (50.8) | | | 0.005 | 0.007 |
| D6S2746 | 10/81 (12.3) | 30/81 (37) | | | **0.003** | **0.006** |
| D6S2447 | 11/86 (12.8) | 40/86 (46.5) | | | **0.001** | **0.004** |
|  |  |  | | |  |  |
| **Multivariate logistic regression analyses** | |  | | |  |  |
| **HIV-1 status** | |  | | |  |  |
| **DNA marker** | HIV-1-seronegative | HIV-1-positive | | |  |  |
| D6S2666 | 35/97 (36.1) | 62/97 (63.9) | | | **0.021** | 0.063 |
| D6S2881 | 35/81 (43.2) | 46/81 (56.8) | | | **0.025** | 0.15 |
| D6S2746 | 51/107 (47.7) | 56/107 (52.3) | | | **<0.0001** | <0.001 |
| D6S2447 | 62/109 (56.9) | 47/109 (43.1) | | | **0.002** | **0.012** |
|  |  |  | | |  |  |
| **Tumour stage** (D6S2666) |  |  | | |  |  |
| CIN 1 & 2 | 44/ 97(45.4) |  | | | **0.027** | 0.054 |
| CIN 3 | 25/97 (25.8) |  | | |  |  |
| Invasive | 28/97 (28.9) |  | | |  |  |
|  |  |  | | |  |  |
| **Histopathology** (D6S2666) |  |  | | |  |  |
| Mild dysplasia | 16/97 (16.5) |  | | | **0.015** | 0.09 |
| Moderate dysplasia | 28/97 (28.9) |  | | |  |  |
| Carcinoma *In situ* | 26/97 (26.8) |  | | |  |  |
| Squamous cell carcinoma | 23/97 (23.7) |  | | |  |  |
| Adeno-squamous cell carcinoma | 4/97 (4.1) |  | | |  |  |
|  |  |  | | |  |  |
| **ROC curves analyses for age** |  |  | | |  |  |
|  | **LOH/MSI** | | | |  |  |
| **DNA marker** | **HIV-1-seronegative** | **HIV-1-positive** | | |  |  |
| D6S2666 | AUC=0.8281 p=0.005, q=0.005 | | AUC=0.7348  **p=0.01, q=0.02** |  | |  |
| D6S2881 | AUC=0.9615 **p=0.042**, q=0.083 | | AUC=0.6545 p=0.1354, q=0.1354 | | |  |
| D6S2746 | AUC=0.5577 p=0.7989, q=0.7989 | | AUC=0.6328 p=0.1760, q=0.352 | |  |  |
| D6S2447 | AUC=0.8611 p=0.1015, q=0.1015 | | AUC=0.7063 **p=0.0224**, **q=0.044** | |  |  |

Where; AUC=Area under the curve
